# The Hospital

**Published:** 1918-02-02

**Authors:** 


THE
### INSTITUTIONAL WORKER
**Being a Special Supplement to "The Hospital"**

### OUR BUREAU OF INFORMATION.

### Rules for Correspondents.

1. Every letter must be accompanied by the coupon to be cut from the back cover (inside page) of THE HOSPITAL, current issue, and must contain the name and address of the correspondent with pseudonym for publication if desired. Replies by post cannot be given save under exceptional circumstances at the Editor's discretion.

2. Letters from Approved Homes in reply to special needs published in the Bureau should state terms and full particulars, and be sent prepaid under cover to the Editor of the Bureau with name written across coupon for identification.

3. Proprietors of Homes which have not yet been entered on the List of Approved Homes, but have spare accommodation likely to suit special needs, are invited to write for an application form for registration. The fee for registration, which includes two announcements of the Home in the Bureau and other privileges, is 10s.

4. All communications to be addressed to the Editor of THE HOSPITAL, 28 Southampton Street, Strand, London, W.C. 2, and marked "Bureau of Information."

### MILK SUPPLY AND DISTRIBUTION.

### Municipal and Other Schemes.

HOSPITALS and other similar institutions where provision has to be made for a large number of inmates are experiencing serious difficulties in consequence of the scarcity and high prices of some of the most necessary articles of diet. The shortage of milk throughout the country is being felt acutely in the hospitals. The latest issue of Lord Rhondda's *National Food Journal* mentions, in commenting on this question, that one important municipality has found itself compelled to pay the retail price for milk for the institutions under its charge, with the result that the expenses have been increased by £10,000 a year. In other cases difficulty has been experienced in securing milk in sufficient quantities even at the retail price.

It should be known by those responsible for hospital administration that under the Milk (Amendment) Order, 1917, it is provided that in sales to institutions by any person, whether he be the producer of the milk or not, in quantities of not less than seventeen Imperial gallons to be delivered in any one day, the maximum price shall be at the rate of 2s. 2d. per Imperial gallon, including all charges for delivery to the buyer's premises, or the maximum retail price for the time being in force in the area in which the institution is situated, whichever be the less.

NORWICH MUNICIPAL SCHEME.

In view of the importance of the problem, the Norwich Public Health Committee have devised what they consider an ideal municipal scheme of milk supply. They suggest the acquisition by the Corporation of the necessary milk for the city direct from the producers, and that its distribution be carried out by licensed retailers in prescribed areas and subject to regulations securing the purity and cleanliness of the milk. Meanwhile, the proposal is to map out the city into ninety-four districts, each containing about 300 houses and 1,100 people, one retailer, licensed under the consent of the Food Controller, to be given the exclusive right to supply a particular district. Hospitals and other institutions which have in existence separate contracts are for the time being excluded from the scheme.

SUPPLY FOR MOTHERS, INVALIDS, AND YOUNG CHILDREN.

With regard to schemes for supplying milk to young children—particularly those of the poorer section of the community—that of the Lewisham Public Health Committee may be cited as somewhat of a model. Its intention is to secure priority in the supply of fresh milk to mothers, invalids, and young children, and, in the case of necessitous persons, to provide it at a reduced price, or even free. The medical officer of health considers that a sufficiently wide interpretation can be placed upon the word "necessitous" to enable him, through the health visitors and the infant-welfare centres, to supply all those really needing milk and unable to obtain it through the ordinary channels. Applications for milk have to be signed by the medical officer of health, the medical officer of one of the welfare centres, or by a general practitioner. Cards showing the weekly quantity of milk allocated to applicants will be issued, and these must be presented by the applicants to dairymen chosen by the Health Committee. The milk must be fetched from the dairymen's premises, and the quantity supplied will be marked on the card. Lewisham's excellent infant-welfare organisation—consisting of six whole-time health visitors and five voluntary infant-welfare centres working under the supervision of the medical officer of health—will no doubt materially assist in the success of the scheme. Every inducement will be offered to mothers receiving milk for their children to bring them to the infant-welfare centre, where their health and progress will be watched.

LORD RHONDDA'S MODEL PRIORITY SCHEME.

All health authorities are being asked to adopt Lord Rhondda's model priority scheme for the supply of milk to young children. Under this scheme priority tickets will be issued only to the following classes: (1) Children up to five years of age; (2) persons holding a qualified medical practitioner's certificate that they are entitled, for health reasons, to a certain quantity of milk per day; (3) any class of persons medically recommended. The amount of milk supplied will be (1) For children—not more than one and a half pints a day if under eighteen months of age, and one pint daily if between eighteen months and five years, and (2) for adults—the quantity specified on their certificate, or generally recommended for the class to which they belong. This scale, it should be noted, is based on the recommendations of the Astor Committee on the Production and Distribution of Milk, which had before it the views of a committee of the Royal Society on the quantity of milk requisite for children of different ages. The priority tickets will not be available for the purchase of milk after 10 A.M., unless the supply has been ordered and paid for in advance. Priority schemes on these lines should now be in force all over the country.

### FEBRUARY QUESTIONS.

### (1) Hot Dinner-Plates.

A hospital has four wards, each containing twenty beds. The dinners are served from one inadequate general kitchen and there is no arrangement for heating the plates. Each ward has a very tiny kitchen, which holds a small gas-ring. How can the plates be best heated?

### (2) How to Sterilise Rubber Gloves.

Describe the best method of sterilising rubber gloves, to be used during operations, to ensure their being perfectly sterilised with the minimum amount of damage to the rubber.

### RULES.

The following rules must be observed:—

1. Contributions must be written on one side of the paper. Brevity and terseness are desirable features. The MSS. must bear the name and address of the sender and be accompanied by coupon to be cut from the back cover (inside page) of the current issue of The Hospital. A pseudonym must be chosen if the name is not to be published.
2. Contributions must be addressed to the Editor of The Hospital, Institutional Worker Supplement, 28 & 29 Southampton Street, Strand, London, W.C. 2, should reach him before the end of the current month, and be marked in the left-hand corner "Facts and Figures."

**A minimum payment of Five Shillings will be made for each published answer to the January Questions.**

### QUESTIONS INVITED.

In connection with our Question Box, we offer every month five shillings each for the two best questions which are sent in for consideration. The questions may be on any institutional subject and concern any department, from the secretary's to the porter's, or the matron's to the domestic staff; the one essential is that they must be practical and deal with points that have a definite relation to hospital or institutional administration, upkeep, finance, or management. Points relating to artificers' work, housekeeping, the laundry, out-patients, and other practical matters will be welcome. It is hoped that thus, when difficult or doubtful points arise in the course of their work, institutional workers will be encouraged to put them in the form of a question and send them to the Editor, The Hospital Bureau of Information, 28 & 29 Southampton Street, Strand, W.C. 2, marked "Question Box."

The best questions will be published in due course and our readers will be given the opportunity of answering them. Thus all institutional workers may be able to cooperate with the view of helping the work and smoothing the difficulties of each other. In short, we want the Question Box of the *Institutional Worker* to be freely used by every worker habitually.

### ENQUIRIES AND ANSWERS.

### *THE SICK AND IN NEED.*

### Heart Case.

One of the general hospitals in London might receive this case of dilated heart, either Guy's, the Royal Free, the London, or St. Bartholomew's, and we suggest that you write to the secretary of each institution, stating the facts of the case. If the patient could afford to make a small payment weekly, she might be admitted to the National Hospital for Diseases of the Heart, Soho Sqare, W.—B. K. R.

### Convalescent Nurse.

If, as you say, you can afford to pay a small weekly sum, we suggest that you apply to Queen Mary's Coronation Home, Whitstable, Kent.—Nurse.

### *EMPLOYMENT AND TRAINING.*

### Short Training in Practical Nursing.

It would be quite impossible for you to gain an insight into practical nursing which would be of any value to you in a month. There are training-schools which take paying pupils for a period of three months, and we give you the names of some of these: The London Hospital, London, E.; St. Bartholomew's Hospital, West Smithfield, E.C.; Guy's Hospital, London, S.E., and St. Mary's Hospital accept paying probationers for twelve months' training.—Isa.

### District Nursing.

A three years' certificate from a general hospital or a Poor-Law infirmary is advisable if you wish to become a district nurse under the direction of Queen Victoria's Jubilee Institute for Nurses, but mixed training is advisable. If, upon application, you are considered suitable, you will receive six months' free training in district work; at the end of six months, after passing a simple examination and one month's trial, you will, if approved, be required to sign an agreement to work for one year as a district nurse wherever required. The salary paid during the six months' training is at the rate of £30 per annum. Applications should be addressed to The General Superintendent, 58 Victoria Street, London, S.W.—C. C.

### *MISCELLANEOUS.*

### Prepared Wax for Floor Polishing.

You will find Johnson's Prepared Wax a most satisfactory floor-polisher and cleaner. It contains no oil, therefore it does not collect and hold dust. This wax is a disinfectant, and is also very economical, as floors need only be polished two or three times a year. The price is 2s. 9d. a lb. if bought in 100 lots and 2s. 4d. if bought in 200 lots, and can be obtained either in 1, 2, or 4 lb. sizes. The same firm also prepare an excellent cleaner for badly stained and greasy surfaces. The address is Messrs. S. C. Johnson and Son, 244 High Holborn, London, W.C. 1.—A. S. U.

### Distemper for Walls.

Paper as a covering for walls in an institution of the sort you mention is not suitable. The question of the best method of treating the walls depends largely on the condition of the plaster when the walls have been stripped. If they show cracks and have an uneven surface paint would not look well, but if they are in good condition paint would be the most suitable, though, of course, more expensive. Failing paint, a distemper, such as Hall's Distemper, is found to be quite satisfactory. There are various other distempers on the market, amongst them being Chambers' Corossos Washable Distemper (T. F. Chambers & Co., Ltd., Kensington Works, Oxford Street, Hull). Washable distempers are found to be preferable. If the builder makes the distemper he should use some fixing material with it. If the plaster is in good condition, a satisfactory plan, especially where there are children, is to paint the walls dado high (4 ft. or 4 ft. 6 in.), and distemper the upper part, the dado being of a darker shade.—A. M. B.

### *WAR MATTERS.*

### Address of the Australian Red Cross Society.

The new address of the Australian Red Cross Society offices is 36 Grosvenor Place, London, S.W. 1.—Red Wing.

### Red Cross Club in Edinburgh.

Would you care to become a member of the club at 8 Hope Street, Edinburgh, which offers a rest-place for Red Cross workers in that city who are without friends with whom they can spend their spare time? Amongst the many privileges the club offers is the use of the bathroom, where hair can be washed and dried in an adjoining bedroom by a gas-fire. We suggest that you either write to or call upon the Secretary for further particulars.—Lonely One.

### The Red Cross Journal.

"The Red Cross," the official Journal of the British Red Cross Society, can be obtained direct from the Society, 83 Pall Mall, London, S.W. 1, for 2s. per annum, including postage. Single copies are threepence.—Member.

### American War Relief Committee.

The address of the American War Relief Committee is The American Embassy, London. The chairman is Mrs. Dickson. The committee looks after stranded Americans and repatriates any who might become a charge on this country. We suggest that you write to the Secretary, giving full particulars of your case.—American.

